# Cyclosporine A rescues the influenza virus fusion with IFITM3-expressing cells by relocating the restriction factor to intraluminal vesicles of multivesicular bodies

**DOI:** 10.1128/jvi.02045-25

**Published:** 2026-03-25

**Authors:** Sergey A. Buth, Mariana Marin, You Zhang, Ori Avinoam, Gregory B. Melikyan

**Affiliations:** 1 Department of Pediatrics, Division of Infectious Diseases, Emory University School of Medicine 12239, Atlanta, Georgia, USA; 2 Children’s Healthcare of Atlanta, Atlanta, Georgia, USA; 3 Department of Biomolecular Sciences, Weizmann Institute of Science 34976, Rehovot, Israel; University Medical Center Freiburg, Freiburg, Germany

**Keywords:** influenza virus, viral fusion, hemifusion, multivesicular bodies, intraluminal vesicles, IFITM3, cyclosporine A

## Abstract

Interferon-induced transmembrane protein 3 (IFITM3) blocks fusion of many enveloped viruses with endosomes, likely by rigidifying the cell membrane. We and others have previously reported that cyclosporine A (CsA) treatment rescues the ability of the influenza A virus (IAV) to fuse with and infect IFITM3-expressing cells, but the mechanism of such rescue remained controversial. Here, we employed correlative light-electron microscopy (CLEM) and on-section electron tomography workflow to visualize the IAV fusion intermediates arrested by IFITM3. Most common intermediates were tight IAV contacts with the limiting membrane and intraluminal vesicles of late endosomes. A hemifusion intermediate was also detected. We further visualized the effect of CsA on IFITM3 localization by immunoelectron microscopy (immuno-EM), which revealed a marked relocation of IFITM3 from the limiting membrane to intraluminal vesicles of endosomes. This CsA-induced IFITM3 sequestration in intraluminal vesicles likely relieves the block for IAV fusion with the limiting membrane of endosomes that culminates in a release of the viral nucleoprotein complex into the cytoplasm.

**IMPORTANCE:**

The mechanism by which interferon-induced transmembrane protein 3 (IFITM3) inhibits the fusion of diverse enveloped viruses with endosomes is not fully understood. Using correlative light-electron microscopy (CLEM) and on-section electron tomography, we detected IFITM3-arrested influenza A virus fusion intermediates with the limiting membrane and intraluminal vesicles of late endosomes. The most common arrested intermediate was a tight contact between the viral membrane and the limiting membrane of late endosomes, which formed more frequently compared to tight contacts with intraluminal vesicles. Immunoelectron microscopy (immuno-EM) revealed that pretreatment of IFITM3-expressing cells with cyclosporine A induced relocation of IFITM3 from the limiting membrane to intraluminal vesicles of endosomes and rescued the influenza A virus fusion. Collectively, these findings imply that the influenza virus fuses with the limiting membrane of endosomes, but not with intraluminal vesicles, leading to productive infection, and that IFITM3 enrichment at these sites is critical for blocking viral fusion.

## INTRODUCTION

Human interferon-induced transmembrane proteins, IFITM1, -2 and -3, are small proteins that exhibit broad antiviral activity against many viruses, including the influenza A virus (IAV), West Nile virus, respiratory syncytial virus, Ebola virus, and HIV-1 (1–9). IFITM2 and -3 predominantly localize to late endosomes (10, 11), whereas IFITM1, which lacks the N-terminal internalization signal, is mostly localized to the plasma membrane (7, 12). The subcellular distribution of IFITMs appears to be the major determinant of the spectrum of restricted viruses, with IFITM3 being more potent against viruses entering from late endosomes (reviewed in references 1, 3). We and others have shown that IFITM3 interferes with the fusion step of virus entry, without blocking the pH-mediated refolding of fusion proteins of diverse viruses entering via endocytosis (1, 2, 4, 10, 11, 13, 14).

The currently accepted “tough membrane” model of IFITM-mediated restriction posits that expression of these proteins prevents viral fusion by rigidifying the cell membranes (3, 4, 15, 16). We have shown that the short amphipathic helix within the N-terminal segment of IFITM3 is necessary and sufficient to render cellular and model membranes more rigid (16). Our single IAV tracking experiments in live cells revealed potent inhibition of viral fusion, without affecting virus hemifusion (lipid mixing) (13, 17). Collectively, these findings demonstrate that IFITM3 arrests IAV fusion at a hemifusion stage. Importantly, a recent cryo-electron tomography (cryo-ET) study directly visualized hemifusion structures formed by IAV in IFITM3-expressing cells (18).

We and others have reported that cyclosporines and rapamycin rescue viral entry into IFITM3-expressing cells (19–22), but the mechanism behind this antagonism with IFITM3 remained unclear. Whereas drug-induced degradation of IFITM3 has been reported in other studies (19, 21, 22), our results have not revealed a considerable reduction in the IFITM3 level by cyclosporine A (CsA) on a time scale of IAV entry (20). Instead, we have detected an apparent relocalization of IFTIM3 from endosomes to the Golgi area in CsA-treated and digitonin-permeabilized cells (20). Our recent results suggest that the Golgi-associated pool of IFITM3 represents the newly synthesized protein, whereas the major fraction remains in endosomes but becomes inaccessible to antibodies against the cytoplasmic N-terminus in mildly permeabilized cells (23). We, therefore, hypothesized that CsA-mediated rescue of IAV fusion (20) is achieved through IFITM3 redistribution from the limiting membrane (LM) to intraluminal vesicles (ILVs) of endosomes. Our super-resolution microscopy results (23) revealed a shift in IFITM3 signal from the periphery to the interior of endosomes, supporting CsA-mediated relocalization of this protein to ILVs of late endosomes.

Here, we examined the ultrastructural basis for IFITM3-mediated restriction of IAV entry using a correlative light-electron microscopy (CLEM) approach, which enabled visualization of IAV fusion intermediates arrested by IFITM3. Furthermore, to delineate the mechanism of CsA-mediated rescue of IAV fusion in IFITM3-expressing cells, we employed immunoelectron microscopy (immuno-EM) and found that CsA treatment induces marked relocalization of this protein from LM to ILVs. These results shed light on the mechanism by which CsA relieves the IFITM3-imposed block for IAV fusion with endosomes.

## RESULTS

### IAV fusion with IFITM3-enriched endosomes is arrested at tight membrane contact and a hemifusion stage

**Fig 1:**
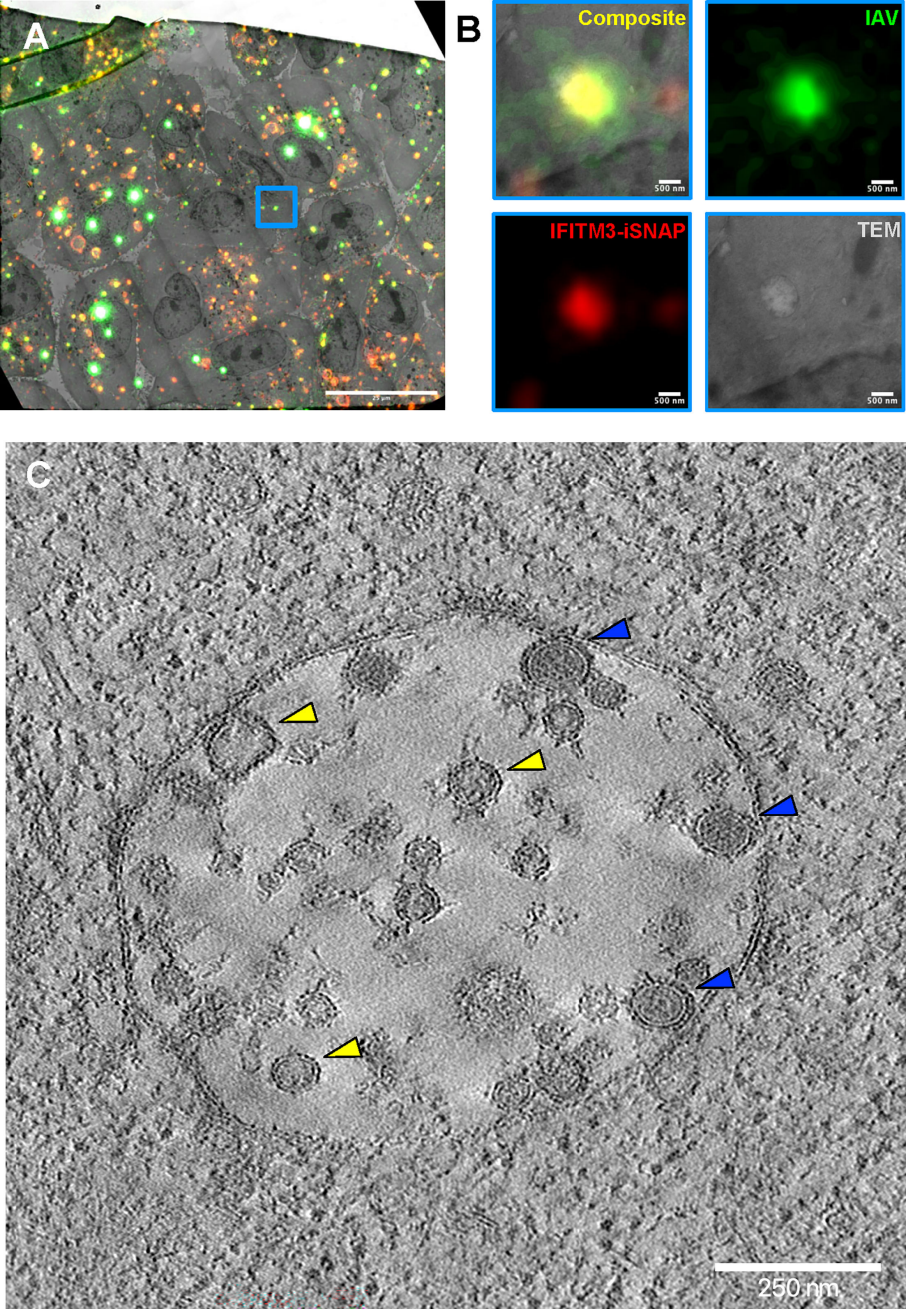
CLEM analysis of the influenza A virus fusion with endosomes in IFITM3-expressing cells. (**A**) CLEM overlay of an individual grid square within a 250 nm-thick section containing A549.IFITM3-iSNAP cells infected with IAV A/PR/8/34 labeled with amine-reactive AF568-NHS dye (green, MOI 1.5) for 1 h. Before infection, IFITM3-iSNAP was labeled with SIR-647 dye (red). Cells were high-pressure frozen, freeze-substituted, and embedded in resin. Resin blocks were sectioned at 250 nm nominal thickness. The 200 nm ex./em. 505/515 nm fluorescent beads added to the sections before fluorescence imaging are colored in magenta (appearing as high intensity green-white puncta: magenta + bleed through into TRITC [colored green] channel = white). An ROI encompassing the selected endosome is marked with a blue square. (**B**) A zoom-in of the overlay and individual channel images of the endosome selected in panel A (blue square). (**C**) A 10 nm-thick slice through a tomographic reconstruction collected at correlated position of the corresponding ROI in panel A. Selected IAVs and ILVs are indicated with blue and yellow arrowheads, respectively. Scale bars in** A, B**, and **C** are 25 µm, 500 nm, and 250 nm, respectively.

Single virus tracking in live cells and a recent cryo-ET study revealed that IFITM3 expression arrests IAV fusion at a hemifusion stage by disfavoring the formation of fusion pores (13, 17, 18). We sought to further characterize the mechanism of IFITM3-mediated restriction of IAV entry using a CLEM approach that enables selective electron tomography studies of IFITM3-positive endosomes that harbor internalized IAV particles (see Materials and Methods). For these experiments, we used A549 cells stably expressing IFITM3 tagged with an “internal” SNAP tag (IFITM3-iSNAP), which exhibits wild-type levels of anti-IAV activity (Fig. S2A and B). Cells were grown on sapphire discs, labeled with a far-red SNAP-reactive dye, and infected for 1 h with IAV labeled with the orange AF568 dye. This infection time was chosen based on the kinetics of IAV fusion with parental A549 cells, which reaches a plateau at 1 h (Fig. S2C). Cells were then subjected to high-pressure freezing, freeze substitution, and embedding in a resin. We then imaged 200–250 nm sections of cells by fluorescence microscopy and transmission electron microscopy (TEM). Endosomes positive for both IAV and IFITM3-iSNAP signals were identified by fluorescent microscopy of thin section (Fig. 1A and B). Selected endosomes were found in TEM by correlating the fluorescence microscopy images with TEM images, followed by the collection of dual-axis tilt series and tomographic reconstruction (Fig. 1B and C and Fig. S1A).

**Fig 2:**
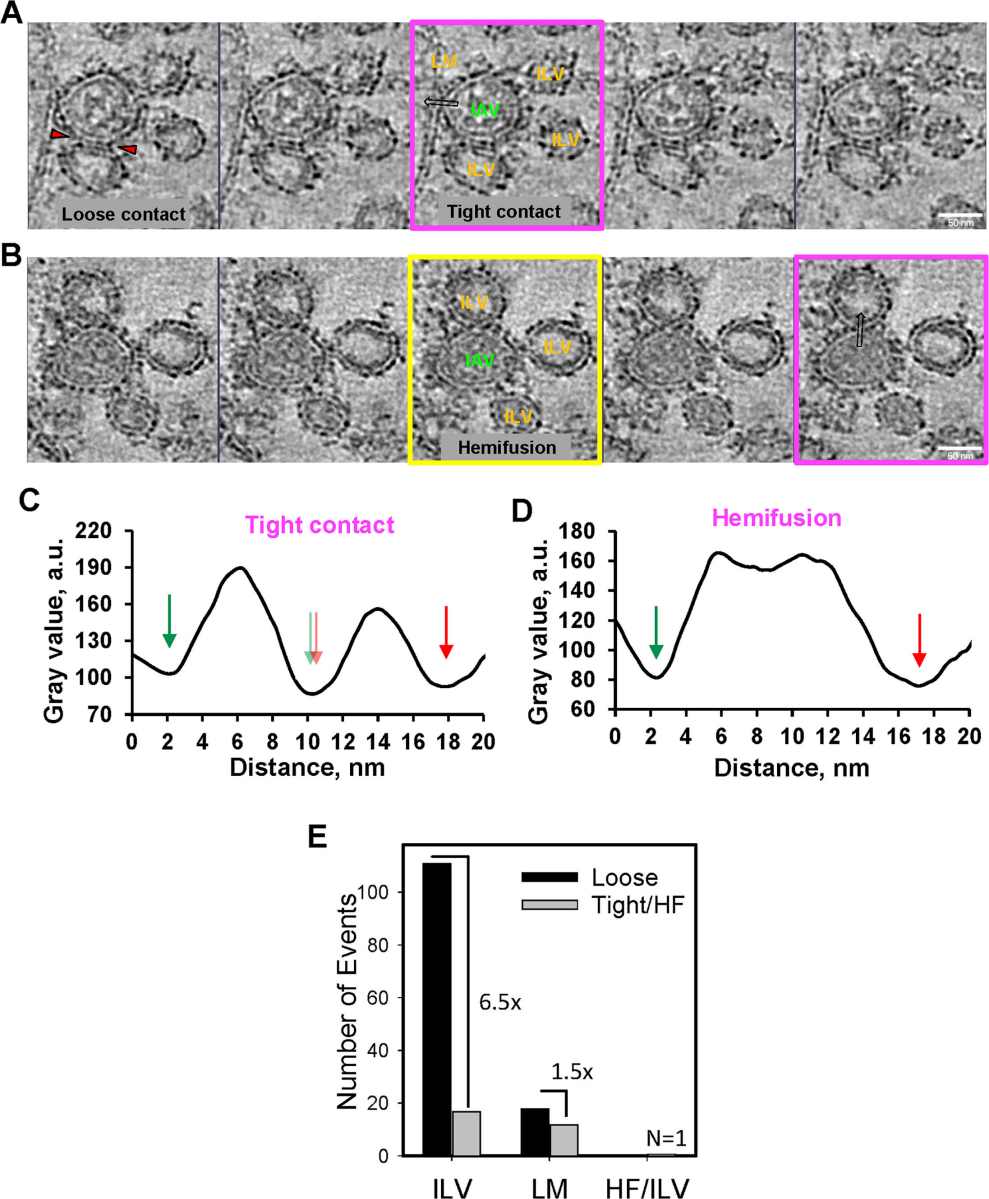
Visualization of the influenza A virus fusion arrested in IFITM3-expressing cells. Consecutive Z slices of the reconstructed tomogram collected at selected endosomes (see Fig. 1), showing the examples of a tight contact between IAV A/PR/8/34 and LM (**A**) and IAV hemifusion with ILV (**B**) of an endosome. Z slices through the tomogram are centered around the tight contact or hemifusion event. Images outlined with magenta were used for 3-pixel wide rectangular profile calculation of IAV-ILV/LM interaction in panels C and D, which are marked with hollow black arrows indicating the profiling direction. The intensity profile through the central slice of the hemifusion affected by a staining artifact blemish in (B, yellow outline) is provided in Fig. S4C. An example of a loose IAV-ILV contact is marked with red arrowheads. (**C**) In the IAV-LM tight contact site, the density corresponding to the contacting and distal membrane leaflets is observed. The minima, corresponding to distal leaflets of IAV and LM, are indicated with green and red arrows, respectively. The contacting leaflets of IAV and LM are tightly juxtaposed (pale green and red arrows, respectively). (**D**) Intensity profile of the IAV-ILV hemifusion site. Only the distal lipid leaflets of hemifused viral and ILV membranes are traceable (indicated with green and red arrows). (**E**) Quantification of IAV-cell membrane contacts and hemifusion. The number of loose and tight contacts between IAV and ILV and LM of endosomes in A549.IFITM3 cells is plotted. A single hemifusion (HF) event is also included.

Tomograms revealed the presence of several IAV particles and ILVs in endosomes/multivesicular bodies of IFITM3-iSNAP-expressing A549 cells (Fig. 1C, blue vs yellow arrowheads, respectively, and Video S1). The IAV particles can be discerned from comparably sized ILVs with reasonable confidence, owing to their higher internal density (lower gray value) and the presence of HA spikes on their surface (Fig. S3). We observed many instances of IAV particles contacting the LM and ILVs of endosomes in IFITM3-iSNAP expressing A549 cells (Fig. 1C, 2A, and C). While we did not detect a full IAV-endosome fusion, as expected, two types of virus-cell membrane contacts, likely representing IFITM3-arrested fusion intermediates, were observed. The predominant virus contacts (~80%) were “loose” contacts where IAV was separated from the cell membranes by a distance comparable with the apparent membrane thickness and often featuring electron densities between two membranes, likely corresponding to HA spikes (red arrowheads in Fig. 2A and Fig. S8). A single IAV particle often formed contacts with multiple ILVs. We also observed tight contacts between IAV and ILV or LM, which we defined as closely abutted viral and cellular membranes, with no detectable gaps (Fig. 2A; Fig. S4A and S8, and Video S2). Most loose and tight contacts formed by IAV were with ILVs (Fig. 2A and E). Notably, however, the fraction of tight contacts with LMs relative to loose contacts was considerably higher than with ILVs (Fig. 2E). Analysis of 10 endosomes in 7 cells revealed a single local hemifusion structure between IAV and ILV (Fig. 2B and Video S3). The *x-z*, *x-y,* and *y-z* sections of the tomogram shown in Fig. 2B illustrate the formation of a local hemifusion structure and contacts between IAV and cell membranes (Fig. S4B).

To clearly discern an IAV hemifusion structure from tight membrane contacts, we analyzed the linear intensity profiles using rectangular ROIs drawn across the membrane contact/hemifusion areas (black hollow arrows in Fig. 2A and B). As expected, juxtaposed membranes showed a clear intensity minimum (referred to as “trough”) separating the two peaks corresponding to the hydrophobic milieu of contacting lipid bilayers (Fig. 2C). By contrast, the linear intensity profile through the hemifusion site exhibited a broad and continuous peak corresponding to a shared membrane (Fig. 2D). These intensity profiles allow for estimation of the apparent bilayer thickness based upon a trough-to-trough distance, D_tt_ (see reference 24 and Fig. 2C and D and Fig. S5).

We note that the apparent thickness of viral and cellular membranes in our heavy metal-stained and resin-embedded samples was 7–8 nm (Fig. 2C and D and Fig. S5), which is nearly twofold greater than the bilayer thickness determined by cryo-EM (18, 25, 26) and other techniques (27). We surmise that the increase in apparent membrane thickness in our samples is due to sample preparation (fixation/dehydration/resin embedding/contrasting) that can introduce structural distortions and complicate the classification of intermediates, such as hemifusion or membrane stacking. Additionally, the high defocus setting (–8 µm) introduces Fresnel fringes, which may artificially inflate membrane thickness or obscure edge definition. It is important that the apparent thickness of a hemifusion diaphragm (14.5 nm) was almost twice the thickness of the IAV and ILV membranes (Fig. 2C and D and Fig. S4). This result is consistent with the formation of a lipid “stalk”—an intermediate structure preceding the abutment of the distal membrane leaflets in a hemifusion diaphragm (28–30).

### CsA redistributes IFITM3 from the limiting membrane of endosomes to intraluminal vesicles and rescues IAV fusion with IFITM3-expressing cells

Our super-resolution microscopy results and biochemical assays imply that CsA induces a relatively quick and nearly complete redistribution of IFITM1 and IFITM3 from the plasma membrane and the limiting membrane of late endosomes, respectively, to the interior of late endosomes (23). We hypothesized that such a relocalization of IFITMs is the likely mechanism of viral fusion rescue by CsA observed by us and others (19–22). To gain ultrastructural insights into the impact of CsA on subcellular distribution of IFITM3, we visualized the IFITM3 localization by immunogold labeling in ultrathin sections.

**Fig 3:**
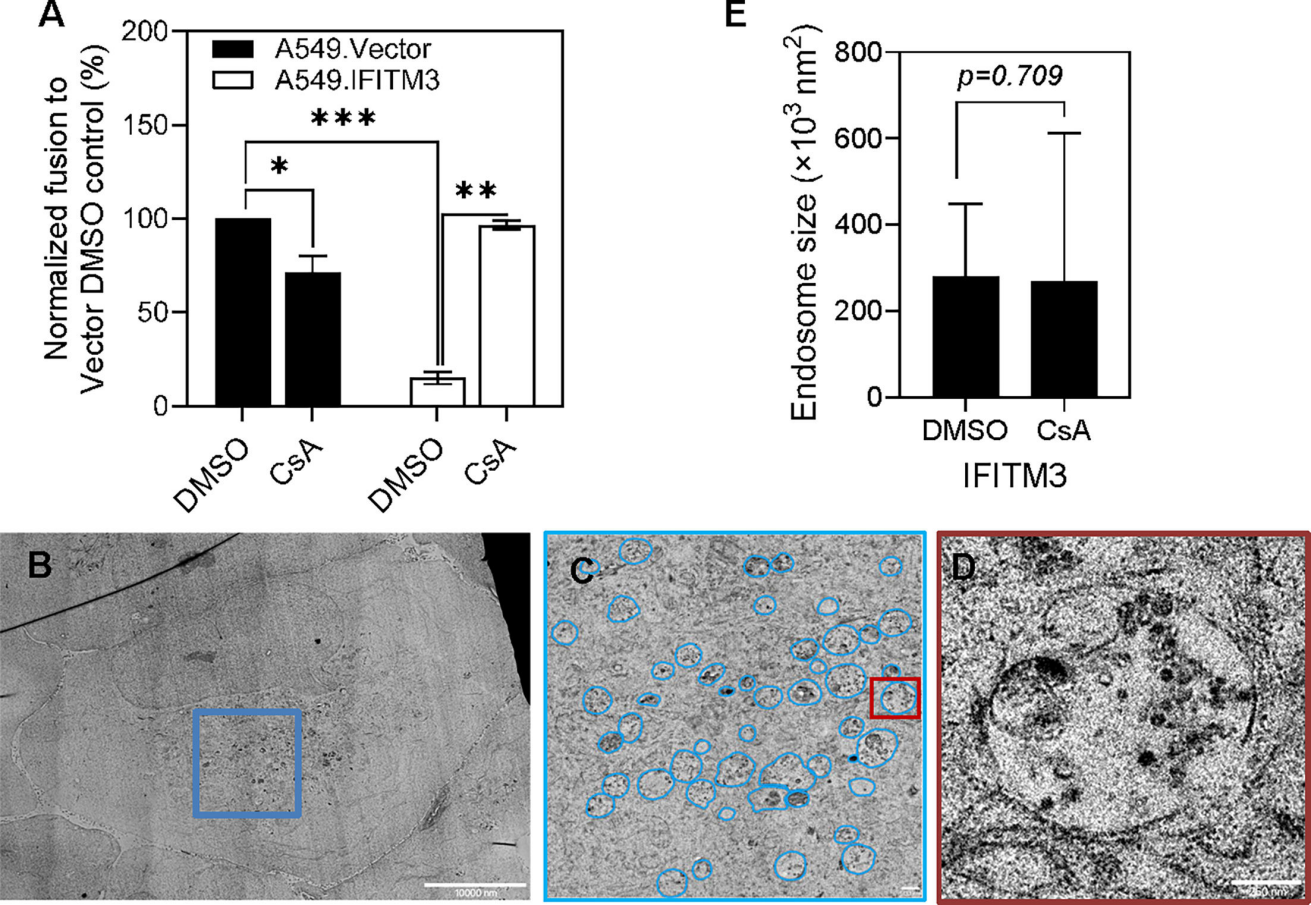
Rescue of IAV fusion with IFITM3-expressing cells by CsA and analysis of endosome size distributions in control and CsA-treated cells. (**A**) A549.Vector and A549.IFITM3 cells were treated either with DMSO or 20 µM CsA for 90 min before infection with IAV pseudoviruses containing BlaM-Vpr. Data are means and SD of two independent experiments, each done in triplicate. Statistical analysis was done using an unpaired *t*-test. *P < 0.05; **P < 0.01; ***P < 0.001. (**B–D**) A549.IFITM3 cells pretreated with DMSO or 20 µM CsA for 90 min were high-pressure frozen, freeze-substituted, resin-embedded, ultrathin sectioned, and imaged by TEM. The endosome size distribution within an endosome-rich perinuclear ~11 × 11 µm cytoplasmic region of interest (blue square) was analyzed. (**B**) An example of a DMSO-treated A549.IFITM3 cell used for endosome size analysis. (**C**) A zoom-in of the blue square area in panel **A**. Endosome contours are outlined in blue. (**D**) A zoomed-in dark red area in panel B featuring a single endosome. (**E**) The endosome size distribution was analyzed by calculating the area encompassed by the LM of individual endosomes. Overall, 201 and 136 endosomes for DMSO and CsA-treated samples were analyzed, respectively, across 5 cells per condition. Statistical significance was determined using Student’s *t*-test assuming equal variances in two samples; the *P* value is based on a two-tailed distribution. See also Fig. S6. Scale bars in panels **B**, **C**, and **D** are 10,000, 1,000, and 250 nm, respectively.

We first confirmed the potent rescue of IAV pseudovirus fusion with A549.IFITM3 cells by CsA pretreatment using a direct virus-cell fusion assay (Fig. 3A). Next, we analyzed the effect of CsA on endosome morphology in A549.IFITM3 or control cells by pretreating these with 20 µM CsA (or DMSO) for 90 min—a condition that effectively rescues IAV fusion (Fig. 3A and [20]). Cells were then subjected to high-pressure freezing, followed by freeze substitution, embedding into antigenicity-preserving Lowicryl HM20 resin, sectioning, and examination of sections by TEM. A comparison of endosome morphology from endosome-rich perinuclear areas of A549.IFITM3 cells did not reveal a significant effect of CsA on the average size of endosomes (Fig. 3B through E and Fig. S6). This result indicates that CsA treatment does not induce the formation of aberrantly sized endosomes and, thus, may not grossly disrupt IAV trafficking and delivery into late endosomes of infected cells where viral fusion occurs.

**Fig 4:**
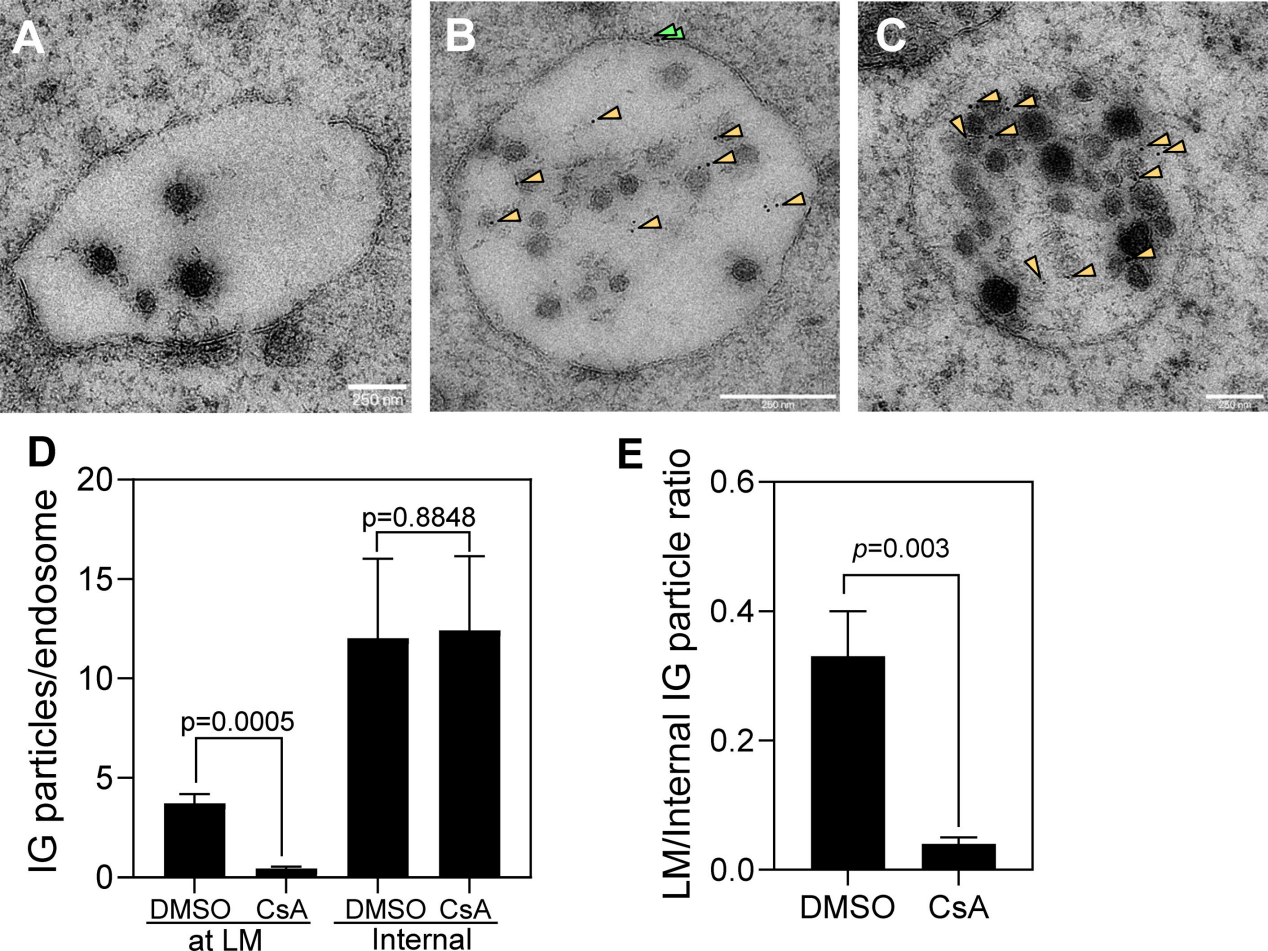
Immuno-EM analysis of the effect of CsA on IFITM3 localization in endosomes. Ultrathin sections containing A549.Vector cells pretreated with DMSO (**A**) and A549.IFITM3 cells pretreated with DMSO (**B**) or CsA (**C**) were stained with immunogold against IFITM3 and imaged by TEM. Gold nanoparticles located within the endosome’s interior and in the vicinity of the LM are marked with orange and green arrowheads, respectively. (**D**) Quantification of gold nanoparticle distribution in experiments shown in panels **B and C** between the LM and endosome interior/ILVs in DMSO and CsA-treated samples. Data are means and SD of blind analyses performed by four independent observers. (**E**) The ratio of LM-associated vs internal gold nanoparticles is calculated from the results in panel **D**. The *P* values using Student’s *t*-test are shown. Scale bar is 250 nm. See also Fig. S7.

To assess subcellular distribution of IFITM3 by immuno-EM, A549.IFITM3 cells were treated with CsA or left untreated, subjected to high-pressure freezing and freeze substitution, and embedded in a resin. Ultrathin sections were incubated with a primary antibody against the N-terminus of IFITM3, followed by labeling with 5 nm colloidal gold-conjugated secondary IgG. Non-specific binding of gold particles to parental A549 cells was low (Fig. 4A and Fig. S7). In contrast, in A549.IFITM3 cells, on average, ~15 immunogold (IG) particles per endosome were detected, in general agreement with reference 18. Most IGs (~70%) were associated with ILVs, while fewer particles colocalized with the LM of endosomes (Fig. 4B and E and Fig. S7). CsA treatment created large intraluminal membrane vesicles, some of which appeared to consist of multiple lamella (Fig. 4C and Fig. S7). Importantly, we observed a dramatic shift of the IFITM3-associated IG particle distribution from the LMs to ILVs of late endosomes in CsA-treated A549.IFITM3 cells (Fig. 4C and D and Fig. S7). CsA treatment markedly reduced the ratio of LM-associated to ILV-associated IG particles by 7–8-fold compared to control cells (Fig. 4E). These findings strongly support our model that CsA induces redistribution of IFITM3 from the limiting membrane to ILVs, thereby removing this protein from the sites of IAV entry and rescuing viral fusion.

## DISCUSSION

Here, we have implemented a robust on-section CLEM workflow for studies of cellular structures and virus fusion with endosomes in 200–250 nm sections of resin-embedded cells. The obtained image quality was sufficient to discern endosomal IAV particles from ILVs and resolve putative intermediates of viral fusion in IFITM3-expressing cells. Nearly all IAV particles were bound to ILVs or LMs, likely through HA glycoproteins (defined as loose contacts), while the minority of virions (30 out of 159) established tight contacts with LM or ILV membranes that likely represented IFITM3-arrested fusion intermediates. Tight contact areas were free of HA spikes and appeared as two closely adjoined membranes. It is possible that such intermediates are stabilized through an increased rigidity of endosomal membranes in IFITM3-expressing cells that disfavors local membrane bending required for hemifusion to occur (16, 31), although similar tight contacts have been detected between IAV and readily deformable liposomes (31). Most of the IAV contacts (loose or tight) in endosomes of IFITM3-expressing cells were with ILVs, suggesting that the abundance of intraluminal vesicles may act as “fusion decoys” in these cells and in control cells lacking the restriction factor. Importantly, the fraction of tight contacts relative to loose contacts was markedly higher with the LMs compared to ILVs (Fig. 2E), perhaps due to the more fusion-favoring properties of the former membranes.

We were able to reliably document only a single IAV hemifusion event in IFITM3-expressing cells. Such a rare hemifusion event contrasts with the recent report of multiple IFITM3-arrested IAV hemifusion structures observed by cryo-ET (18) and efficient lipid mixing (hemifusion) between IAV and endosomes in our live cell experiments (13, 17). Moreover, the hemifusion structure in our CLEM experiments appears to represent an early stage of hemifusion referred to as “stalk,” which precedes the abutment of distal membrane leaflets and formation of a hemifusion diaphragm (Fig. 2B). This observation, along with the larger apparent thicknesses of the viral and cell membranes (Fig. 2 and Fig. S5), may be indicative of disruption and/or masking hemifusion structures upon cell fixation and plastic embedding. This notion is supported by the reversibility of IAV HA-mediated hemifusion under conditions that prevent progression to full fusion (32).

Our immuno-EM experiments provide important insights into IFITM3-mediated IAV restriction and its antagonism by CsA. In agreement with the previous immuno-EM study (10), we detected IFITM3 in both the limiting membrane and intraluminal vesicles of late endosomes. Most IFITM3-bound IG particles were associated with ILVs, also in agreement with (10). CsA treatment redistributed IFITM3 from LM to ILVs (Fig. 4). This critical observation validates our recent super-resolution microscopy results suggesting less peripheral localization of IFITM3 within the late endosomes after CsA treatment (23). The IFITM3 relocalization to ILVs, along with the rescue of IAV fusion with IFITM3-expressing cells after CsA treatment, implies that IAV fusion with LM, but not with ILVs of late endosomes, represents a productive entry pathway. The preferred IAV fusion with LM of endosomes is consistent with the higher fraction of viral tight contacts formed with LM vs ILVs (Fig. 2E). Whether virus-ILV fusion followed by “back-fusion” of the fusion product with LM of endosomes can lead to productive infection is controversial (33, 34). This pathway appears unlikely in the context of IFITM3 expression, which inhibits the back-fusion reaction (35). We therefore favor the idea that ILVs act as “fusion decoys” for virus entry (13).

Presently, the mechanism by which CsA relatively quickly relocates IFITM1 (23) and IFITM3 from the plasma membrane and LM, respectively, to ILVs is not understood. We did not detect clear effects of CsA on IFITM’s post-translational modifications or its ability to reduce infectivity upon incorporation into viral particles (23). Interestingly, CsA treatment induces the formation of large, often multilamellar, membrane inclusions inside late endosomes (Fig. 4 and Fig. S7). These findings are indicative of a global modulation of curvature of the IFITM3-containing LM membrane, resulting in an inward budding that may relocate IFITM3 from the LMs. While our recent super-resolution microscopy results suggest reduction in the diameter of IFITM3-positive endosomes in CsA-treated cells, a global analysis of endosomal morphology in EM images did not show a significant reduction in size after CsA treatment (Fig. 3E). This discrepancy may reflect a differential effect of the drug on the IFITM3-positive pool of endosomes analyzed previously vs the entire endosome population visualized by EM. If the average size of an endosome is unaffected, accumulation of internal lipid structures in CsA-treated cells should be associated with a compensatory lipid influx to the LMs of endosomes. Future correlative cryo-ET studies may shed light on the nature of these CsA-mediated changes in endosome morphology and IFITM relocalization.

## MATERIALS AND METHODS

### Cell lines, plasmids, and reagents

Human HEK293T/17 and A549 cells were obtained from ATCC (Manassas, VA, USA). A549, stably expressing IFITM3, A549.pQCXIP-IFITM3, A549.pQCXIP-IFITM3-iSNAP, and A549.pQCXIP vector cells (negative control) were described previously (13, 16). Cells were cultured at 37°C, 5% CO_2_ in high-glucose Dulbecco’s Modified Eagle’s Medium (DMEM; 10-013-CV, Corning, Mediatech, Manassas, VA, USA) supplemented with 10% heat-inactivated fetal bovine serum (FBS; S11150H; R&D Systems, Flowery Branch, GA, USA) and 100 units/mL penicillin/streptomycin (SV30010, Hyclone, South Logan, UT, USA).

The pR9ΔEnv, pMM310 (BlaM-Vpr), pcRev, and pCAGGS vectors encoding influenza H1N1 WSN HA and NA expression plasmids have been described previously (17). Rabbit anti-IFITM3 antibody (ab109429) was purchased from Abcam (Cambridge, UK). Mouse anti-IAV nucleoprotein antibody, clone AA5H (MCA400), was purchased from BIO-RAD (Hercules, CA, USA). Goat anti-rabbit IgG nanogold-conjugated antibody for immunoelectron microscopy (G7277) was purchased from Millipore-Sigma (Houston, TX, USA). Goat anti-mouse nanogold-conjugated antibody (25133) manufactured by Aurion ImmunoGold Reagents & Accessories (Wageningen, the Netherlands) was purchased from Electron Microscopy Sciences (EMS) (Hatfield, PA, USA). The influenza A NP recombinant rabbit monoclonal antibody MA5-42365 was obtained from ThermoFisher Scientific (Waltham, MA, USA). Goat anti-Rabbit IgG-FITC antibody (F9887) and Alexa Fluor 568 NHS Ester (AF568-NHS) (A20003) were purchased from Millipore-Sigma and ThermoFisher Scientific, respectively. SNAP-Cell 647-Sir (S9102S) was purchased from New England Biolabs (Ipswich, MA, USA). Influenza A/PR/8/34 virus (10100374) was obtained from Charles River Laboratories (Wilmington, MA, USA). The replication-competent mCherry-expressing IAV PR/8/34 was a gift from Dr. Luis Martinez-Sobrido (University of Rochester, USA) (36). Cyclosporin A (30024) was purchased from Millipore-Sigma.

### IAV labeling, purification, titration, and infectivity assay

IAV labeling was carried out as described previously (23). Briefly, 50 µL of IAV (2 mg/mL of total protein) was diluted in a freshly made bicarbonate buffer (pH 9.0) and incubated with 50 µM AF568-NHS in the dark, while vortexing, for 1 h at room temperature. After quenching NHS with Tris-buffer (pH 8.0), viruses were passed through a NAP-5 gel filtration column (Illustra, Danaher Corporation, Washington, D.C., USA), and filtered with a 0.45 µm filter (725-2545, ThermoFisher Scientific). The effect of dye labeling on IAV titer was determined by immunostaining A549 cells infected with varied dilutions of virus using an anti-influenza A NP antibody, as described previously (23).

To address the inhibition of replication-competent IAV by IFITM3 and IFITM3-iSNAP, A549 cells stably expressing IFITM3 or IFITM3-iSNAP, or vector cells (negative control) were seeded at 1 × 10^4^ cells/well in a 96-well black clear bottom. The next day, the cells were infected by spinoculation at 4°C, 1,500 × *g* for 30 min with serial dilutions of mCherry-expressing IAV PR/8/34 prepared in DMEM supplemented with 10% FBS. Unbound virus was removed, cells were washed two times with PBS +/+, and incubated in growth medium for 24 h. Cells were fixed with 2% PFA, stained with 10 µM of Hoechst 33342, and analyzed for mCherry-positive cells with a Cytation 5 Cell Imaging Multimode Reader (BioTek Instruments, Winooski, VT, USA).

### Pseudovirus production and β-lactamase (BlaM) virus-cell fusion assay

Pseudoviruses were generated in HEK293T/17 cells using the JetPRIME transfection reagent (# 114-15, Illkirch-Graffenstaden, France). Cells (~75% confluent in a 10-cm dish) were co-transfected with 4 µg of pR9ΔEnv, 1.5 µg of pMM310 (BlaM-Vpr), 0.5 µg of pcRev, and 2.5 µg each of the IAV envelope glycoprotein-encoding plasmids pCAGGS-WSN-HA and pCAGGS-WSN-NA. At 48 h post-transfection, the culture supernatant containing pseudoviruses was collected, filtered through a 0.45 µm PES membrane (#514-1261, VWR, Radnor, PA, USA), and concentrated 10-fold using the Lenti-X Concentrator (#631232, Takara, San Jose, CA, USA). Virus stock was aliquoted and stored at −80°C. The concentration of viral capsid protein (p24) was quantified by ELISA, as previously described (37).

For the BlaM virus-cell fusion assay, IAV pseudoviruses (0.5–1 ng p24 per well) were added to ~90% confluent target cells seeded in black, clear-bottom 96-well tissue culture plates (#3603, Corning, Kennebunk, ME, USA). Infections were carried out by virus spinoculation at 4°C for 30 min at 1,550 × *g*. Cells were washed, and fusion was allowed to proceed for 2 h at 37°C, after which time, cells were loaded with the CCF4-AM substrate (#K1089, Invitrogen Life Technologies, Carlsbad, CA, USA) and incubated overnight at 12°C. Cytoplasmic BlaM activity, indicative of fusion, was measured as the ratio of blue to green fluorescence using a Synergy HT fluorescence microplate reader (Agilent BioTek, Santa Clara, CA, USA). The kinetics of BlaM virus-cell fusion was analyzed by “time of addition” experiment, in which fusion was stopped at the indicated time points by adding 70 mM NH_4_Cl, that raises the endosomal pH.

### Sample preparation for on-section CLEM

Sapphire discs (405; Engineering Office M. Wohlwend, Sennwald, Switzerland) with overlaying London Finder H15 grids (LF-135; EMS) were sputter-coated with 5–7.5 nm of carbon using Denton Benchtop Turbo Carbon Evaporator (Denton Vacuum, Moorestown, NJ, USA). Carbon-coated sapphire discs were glow-discharged for 1 min at “High” radio frequency settings using Harrick Plasma PDC-32G plasma cleaner (Harrick Plasma, Ithaca, NY, USA), placed into the biosafety cabinet, and sterilized for 20 min under the UV light. Sterilized discs were transferred into a 35 mm microscopy dish (P35G-1.5-25-C; MatTek, Ashland, MA, USA) and submerged in 200 µL of 5 µg/mL fibronectin (F0895; Millipore-Sigma) solution, and incubated overnight at 37°C. The next day, sapphire discs were washed in PBS –/–, placed into a new 35 mm MatTek dish, overlaid with ~1 × 10^5^ A549-IFITM3 cells, and incubated overnight at 37°C in 5% CO_2_. The following morning, the sapphire discs with attached A549-IFITM3 cells were washed in PBS –/–, transferred into a new 35 mm MatTek dish, and inoculated with 100 µL of AF568-labeled IAV (estimated MOI ca. 1.5). Virus suspension was spinoculated onto cells at 4°C, 1,500 × *g* for 30 min. Cells were washed with cold PBS +/+ (20-030-CV; Corning) to remove unbound virus. To label IFITM3-iSNAP, cells were incubated in DMEM/10% FBS supplemented with 3 µM of SNAP-Cell 647-Sir at 37°C, 5% CO_2_ for 30 min. Cells were washed and incubated in fresh DMEM/10% FBS for an additional 30 min to remove unreacted dye. Where indicated, cells were pre-treated with 20 µM CsA for 90 min at 37°C before spinoculation with the IAV virus.

After allowing for virus entry, the samples were transported for 30 min at ambient temperature for high-pressure freezing (HPF). For HPF, sapphire discs were removed from the medium, overlayed with a 50 nm thick gold-plated copper spacer ring (1027, Engineering Office M. Wohlwend) on top of adherent cells, and sandwiched between two hexadecane-treated Type B aluminum specimen carriers (242, Engineering Office M. Wohlwend) inside of the HPF specimen holder (290; Abra Fluid, Windau, Switzerland). Specimens were high-pressure frozen according to HPM 010 (Abra Fluid) standard operating procedure and transferred into liquid nitrogen for storage.

### Freeze substitution, resin embedding, and polymerization

Further sample processing was carried out using freeze substitution and low temperature resin embedding system, EM AFS-2 (Leica Microsystems, Wetzlar, Germany), complemented with freeze substitution processor (EM FSP; Leica Microsystems). Sapphire disc-containing sandwiches were split under the liquid nitrogen, and the discs were transferred to a freeze-substitution medium containing 0.1% uranyl acetate (36406; Alfa Aesar, ThermoFisher Scientific), 2% methanol (047339.AE; ThermoFisher Scientific) in glass-distilled acetone (10015; EMS) tempered at –90°C inside of an AFS2 sample processing chamber. Samples were freeze substituted and embedded in MonoStep Lowicryl HM20 resin (23994; Polysciences, Warrington, PA, USA), according to a modified protocol of Kukulski et al. (38): freeze substitution occurred at –90°C for 12 h, after which time, the temperature was raised to –45°C at a rate of 3.5°C/h, and kept at –45°C for 4 h. The samples were washed three times with acetone (10 min each) and infiltrated with increasing concentrations (10%, 25%, 50%, and 75%; 3 h each) of Lowicryl in acetone, while the temperature was further raised in the latter two steps to –25°C at 3.3°C/h. Subsequently, the acetone-resin mixture was replaced by pure resin for 1 h, the resin was then exchanged three times with incubation for 6, 8, and 10 h, and samples were UV polymerized at –25°C for 48 h. The temperature was then raised to 20°C (3.7°C/h), and UV polymerization continued for an additional 12 h.

### CLEM and electron tomography

Resin-embedded samples were sectioned at 200 or 250 nm nominal thickness using EM UC6 microtome (Leica Microsystems), and sections were placed on individual EM copper grids covered with a continuous 5–6 nm carbon film (CF200-Cu; EMS). Grids with sections facing down were incubated for 10 min on top of 20 μL drops of solution containing 1:8,000 diluted in PBS –/– 200 nm fluorescent beads used as fiducial marker (FluoSpheres, Carboxylate-Modified Microspheres, Excitation/Emission 505/515 nm, F8811; ThermoFisher Scientific). Unbound beads were washed three times with ultrapure water and blotted with filter paper (1001; Danaher Corporation, Washington D.C., USA). For fluorescent data collection, each grid was sandwiched between two 25 mm glass coverslips (#1 thickness, 72196-25; EMS), which were mounted in a ring holder (Attofluor cell chamber A7816; ThermoFisher Scientific).

Fluorescence data from sections were acquired with a *z-*spacing of 400 nm using a wide-field DeltaVision microscope (Leica Microsystems) equipped with UPlanFluo 60x/1.42 NA oil objective (Olympus, Tokyo, Japan) and DAPI, FITC, TRITC, CY5 filter set (Chroma, Bellows Falls, VT, USA). Fluorescence emission was recorded by an EM-CCD camera (Photometrics, Teledyne Vision Solutions, Waterloo, Ontario, Canada). Image stacks were deconvolved and z-projected using SoftWorX software available on a DeltaVision workstation.

EM grids were loaded into a single tilt room temperature grid holder equipped with a high tilt retainer and imaged using JEM-2200FS 200 kV field emission gun transmission electron microscope (JEOL, Tokyo, Japan) equipped with an in-column Omega energy and DE-20 direct electron detector (Direct Electron, San Diego, CA, USA) camera. Full grid overview (atlas) and polygon (grid square) montages were collected at 100× and 3,000× nominal magnification, respectively. For data collection, spot size 3 and objective lens aperture #1 with a diameter of 60 µm were used. Omega energy filter was not employed.

To identify positions of endosomes displaying fluorescence signals from both IFITM3 and IAV for electron tomography data collection, selected polygon montages were acquired and correlated with FLM images of the same regions in ecCLEM v2 plugin of ICY bioimaging software using fluorescent beads as landmark points (39).

Dual-axis tilt series data collection in correlated positions was carried out. Tilt series were acquired at 10,000× nominal magnification (calibrated pixel size of 6.31 Å at the level of the specimen) at negative 8 μm defocus using a DE-20 camera in a movie mode. Tilt series were recorded in −60° to 60° angular range using a bi-directional scheme starting from 0° with a 2° tilt increment. Second-axis tilt series data were collected after physical rotation of the grid by 90°. Data collection was carried out using SerialEM software (40). All movie frames were dark-subtracted and gain-normalized on the DE Server, followed by frame alignment and summing using SerialEM on-the-fly implementation. Tilt series were aligned, and tomograms reconstructed with IMOD (41) employing the Simultaneous Iterative Reconstruction Technique (SIRT) with standard Gaussian radial filtering parameters: 0.4 cutoff and 0.035 falloff. Fifteen SIRT iterations were used. CTF correction was not performed. Reconstructed tomograms and single images were visualized using 3dmod.

### Immunoelectron microscopy

The samples for immuno-EM experiments were prepared similarly to those for CLEM experiments, as described above, with minor modifications: A549.pQCXIP (vector cells), A549.pQCXIP-IFITM3 cells were treated with DMSO, and A549.pQCXIP-IFITM3 cells were treated with CsA. The freeze-substitution medium contained 0.2% uranyl acetate, 4% methanol in glass-distilled acetone, and resin-embedded samples for immuno-EM experiments were sectioned at an 85 nm nominal thickness, and sections were collected on individual EM nickel grids covered with a continuous 5–6 nm carbon film (CF200-Ni; EMS). Immunogold labeling was carried out according to reference 42: grids containing sections were incubated on top of 20 µL drops of blocking buffer (0.1% BSA and 0.01 M glycine in 0.05 M PBS –/–), followed by incubation with primary antibodies against IFITM3 diluted 1:20 in diluting buffer (1% BSA in 0.05 M PBS –/–) for 1 h at room temperature and additional overnight incubation at 4°C. Grids were washed with a blocking buffer and incubated for 1 h at room temperature with 1:20 goat anti-rabbit IgG secondary antibodies conjugated with 5 nm colloidal gold. Grids were washed with PBS –/– and ultrapure water, fixed with 2.5% aqueous glutaraldehyde, washed with ultrapure water, and air dried.

Next, the sections were stained in osmium tetroxide vapor for 8 to 10 h by placing the grids with the sections facing down over the 2 mm holes in the screw cap of a 15 mL tube (Falcon 352196; Corning) filled with 1 mL of 1% osmium tetroxide (19110; EMS) aqueous solution. After osmium vapor staining, grids were incubated on filter paper under vacuum for 30 min at room temperature. Grids were examined using JEOL JEM-2200FS in a configuration described above. Single images of endosomes were recorded at 10,000× nominal magnification at a negative 8 μm defocus using a DE-20 camera in a movie mode. Frames were aligned and summed on-the-fly using SerialEM frame alignment, as described above.

### Endosome size and nanogold distribution analyses

Gold nanoparticles associated with selected endosome images were manually counted for CsA (–) and (+) conditions by four independent observers. For three observers, the samples were blinded. For analysis of endosome size distribution in CsA (–) and (+) samples, polygon montages collected at 3,000× magnification were used. Representative cells were selected for both conditions, and all discernible endosomes within a 10.8 × 10.8 µm area were analyzed by a single observer. Data and statistical analyses were performed using Excel and Prism software (Microsoft Corp., USA, GraphPad Software Inc., USA). Details regarding replicates, statistical test used, exact sample sizes (*n*), what *n* represents, and dispersion and precision measures used can be found in figures and figure legends. *P* < 0.05 was considered significant.

## Data Availability

Reconstructed tomograms were deposited to EMDB and are accessible by the following accession numbers: EMD-75342, EMD-75343, EMD-75344, EMD-75345, EMD-75347, EMD-75348, and EMD-75349.

## SUPPLEMENTAL MATERIAL

Supplemental material jvi.02045-25-s0001.pdf Figures S1 to S8; legends for Videos S1 to S3.

Video S1 jvi.02045-25-s0002.mov Reconstructed tomogram collected at representative endosome in DMSO-treated sample pictured in Fig. 1.

Video S2 jvi.02045-25-s0003.mov A zoomed-in region of a reconstructed tomogram featuring a tight contact between IAV particle and endosomal LM shown in Fig. 2A, S3A, and S4A.

Video S3 jvi.02045-25-s0004.mov A zoomed-in region of a reconstructed tomogram featuring a hemifusion between IAV particle and ILV shown in Fig. 2B, S3B, and S4B.

ASM does not own the copyrights to Supplemental Material that may be linked to, or accessed through, an article. The authors have granted ASM a non-exclusive, world-wide license to publish the Supplemental Material files. Please contact the corresponding author directly for reuse.
