# Supplementary material for: Cyclosporine A rescues the influenza virus fusion with IFITM3-expressing cells by relocating the restriction factor to intraluminal vesicles of multivesicular bodies
Source: J Virol. 2026 Mar 25;100(4):e02045-25. doi: 10.1128/jvi.02045-25 (PMC13098200; doi:10.1128/jvi.02045-25)
Supplement: Supplemental material — Figures S1 to S8; legends for Videos S1 to S3. [file jvi.02045-25-s0001.pdf]

## Supplemental Figure and Video Legends

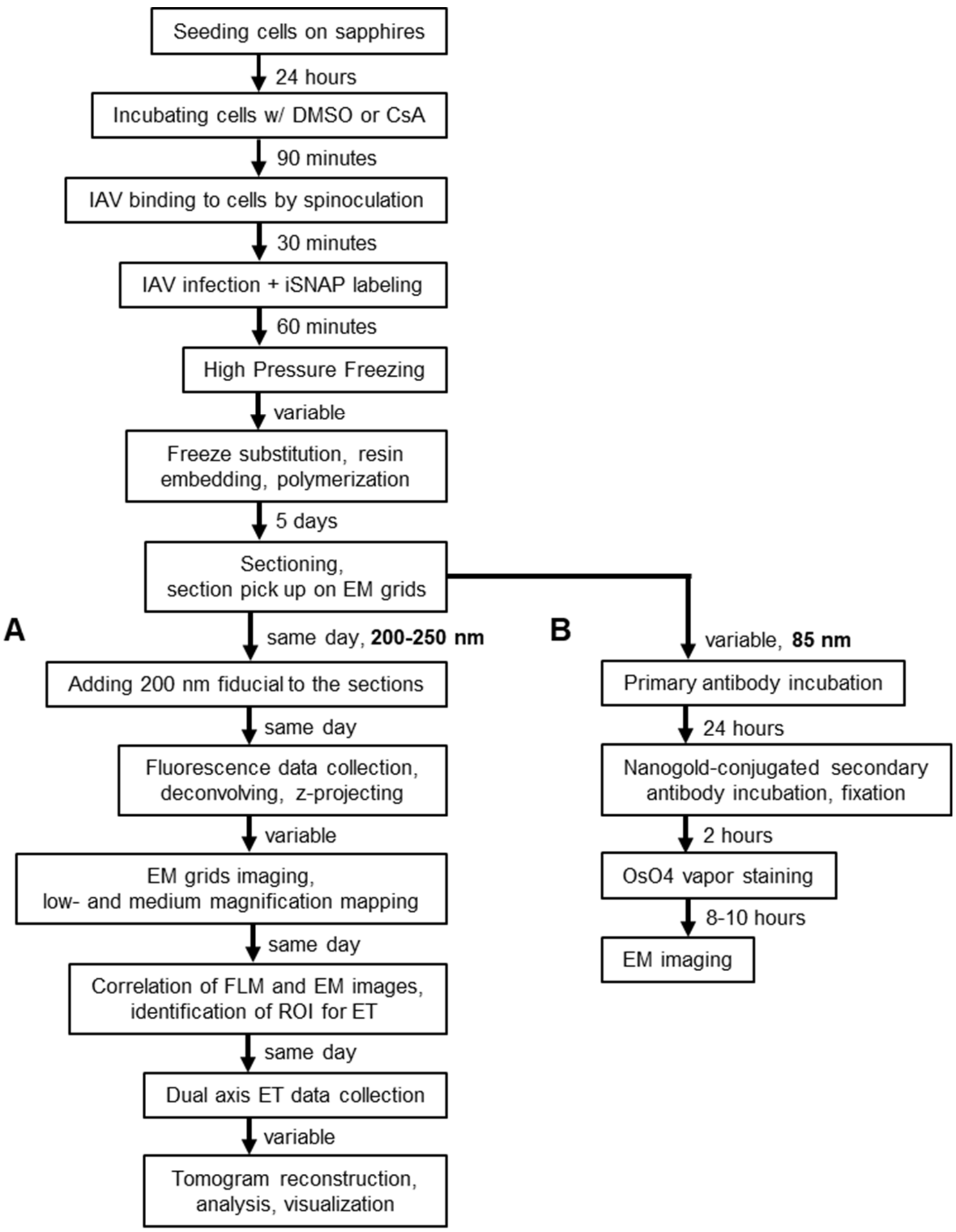


**Supplementary Figure S1.** (**A**) Schematics for on-section CLEM-ET workflow to visualize IFITM3-mediated restriction of virus entry. (**B**) A flow-chart for on-section (post-embedding) immunogold labeling workflow to study intra-endosomal distribution of IFITM3.

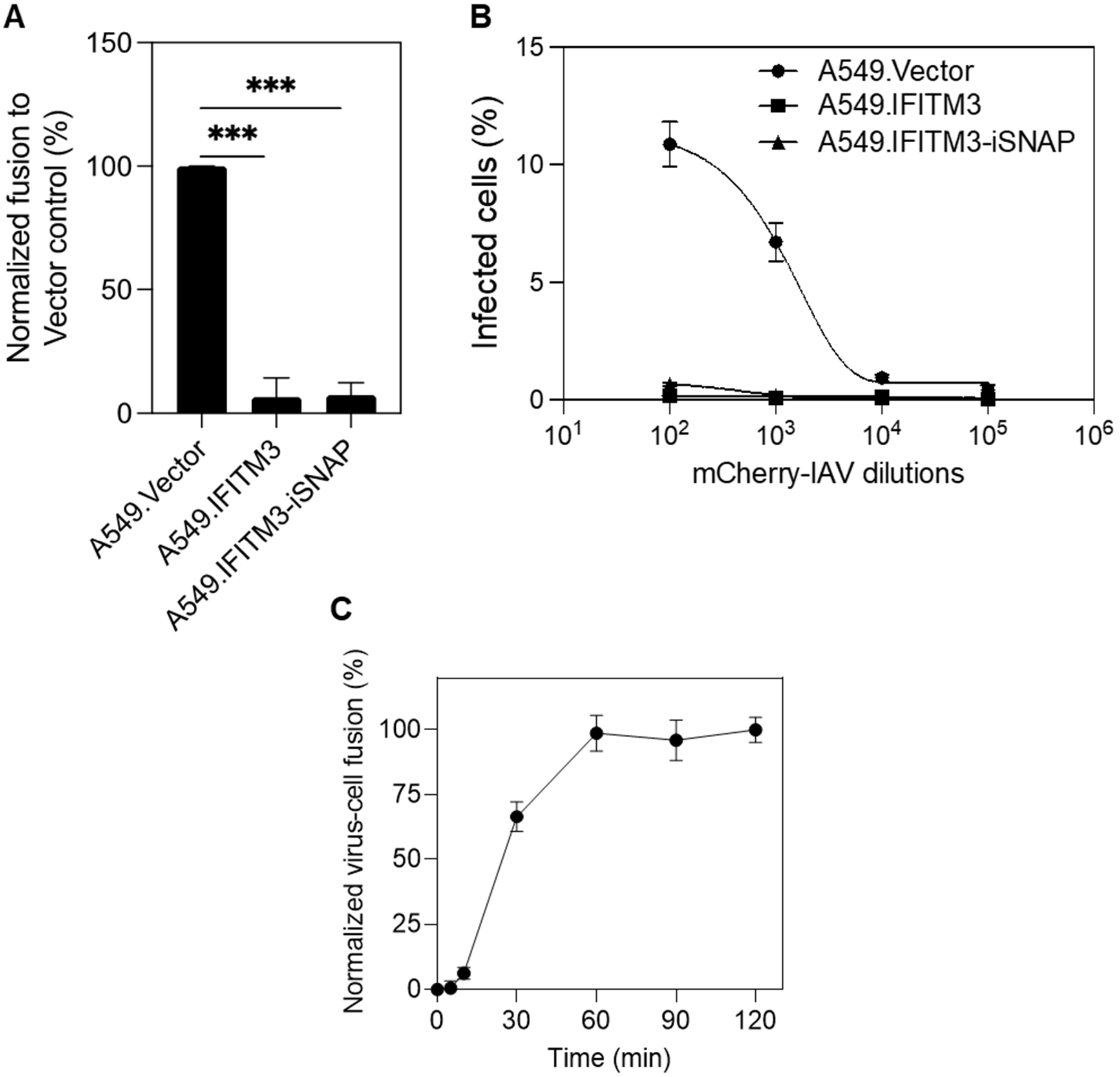


**Supplementary Figure S2. Antiviral activity of IFITM3-iSNAP and the kinetics of IAV fusion with A549 cells.** (**A, B**) A549.Vector, A549.IFITM3, and A549.IFITM3-iSNAP cells were infected either with IAV pseudoviruses containing BlaM-Vpr that allows the measurement of viral fusion (A) or with replication-competent mCherry-expressing IAV (A/PR/8/34 strain) (B). For (A) data are means and SD of three independent experiments, each done in triplicates. For (B) data are means and SD of a representative experiment performed in triplicates. Statistical analysis was done using unpaired t-test. ****p*<0.001. (**C**) IAV/BlaM-Vpr pseudoviruses were bound in the cold to A549 cells for synchronized virus entry. Virus entry/fusion was initiated by shifting to 37 ºC and stopped by adding 70 mM $NH_4Cl$ at varied times of incubation. Data are means and SD of a representative experiment performed in triplicates.

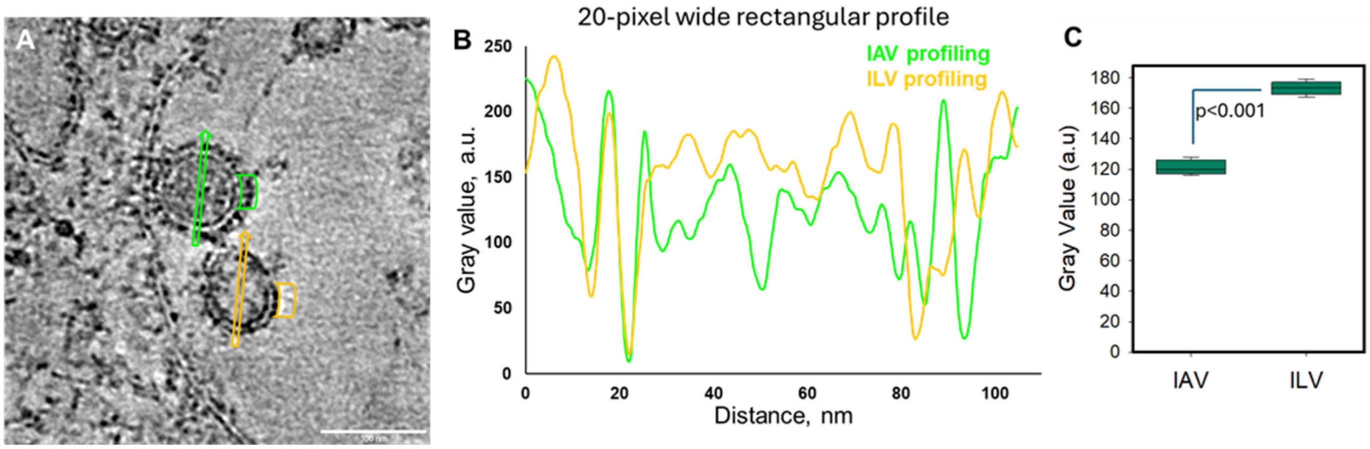


**Supplementary Figure S3. Discrimination between IAV and ILVs. (A)** Tomogram slice featuring IAV (top) and ILV (bottom) particles. Green (IAV) and orange (ILV) hollow arrows indicate 20 pixel-wide rectangular profiling areas, with arrows indicating the direction of profiling. Respective color-matched arcs indicate the HA densities on the surface of IAV and the absence of thereof on the surface of ILV. **(B)** Intensity profiles through IAV (green) and ILV (orange). **(C)** Average gray values for IAV (n=4) and ILV (n=5) interior (Means and SDs are shown).

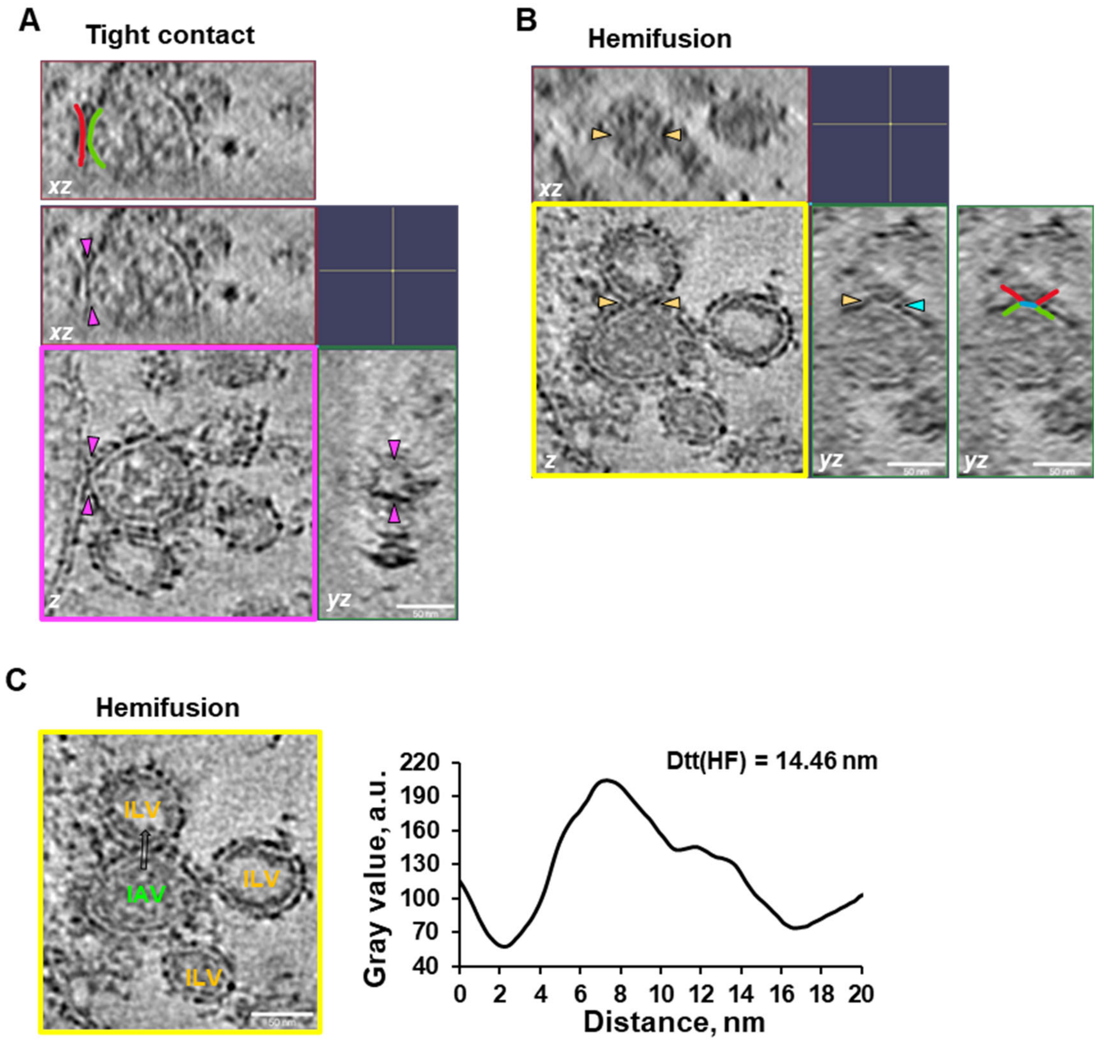


**Supplementary Figure S4.** 3dmod XYZ views of tight IAV-LM contact (**A**) and hemifused IAV/ILV membranes (**B**) are shown. *z*, *xz*, *yz* projections indicated in corresponding views. The area of tight docking and hemifusion is indicated in each projection with magenta and melon arrowheads, respectively. In *yz* projection in (**B**) a post-fusion hemagglutinin glycoprotein seen as an electron dense object perpendicular to the hemifusion diaphragm is indicated with a cyan arrowhead. Additional *yz* and *xz* projections in (**A**) and (**B**) were used to segment viral (green) and ILV/LM (red membranes). Hemifusion diaphragm is segmented with blue. (**C**) Three-pixel rectangular density profile (hollow black arrow) across the central section of the tomogram through the hemifusion site affected by a staining artifact blemish shown in Fig. 2B (yellow outline). The staining artifact blemish at the center of the hemifusion site smears the appearance of the intensity peak corresponding to the shared IAV/ILV membrane. Scale bar is 50 nm.

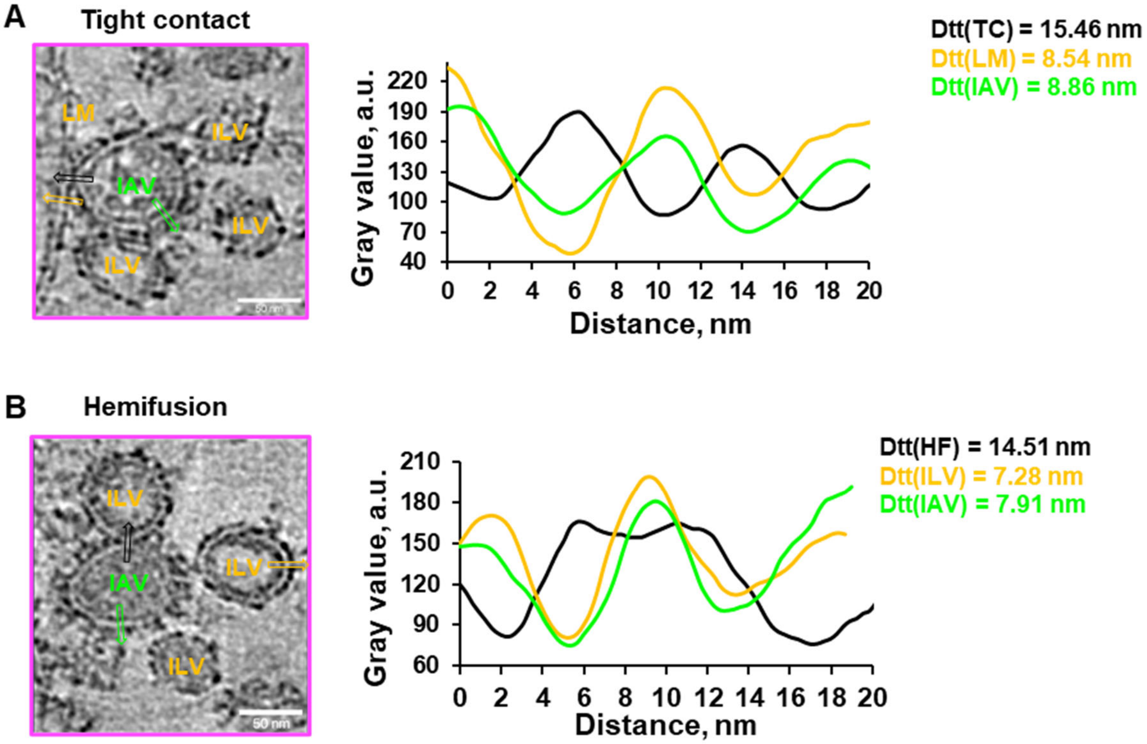


**Supplementary Figure S5.** Analysis of apparent thicknesses of a double membrane forming a tight contact TC (**A**, black) and a hemifusion diaphragm, HF (**B**, black) relative that single membranes of LM (orange in A), ILV (orange in B), and IAV (green). Respective color-matched rectangular histogram profiles for hollow arrows in panels A and B are plotted on the right.

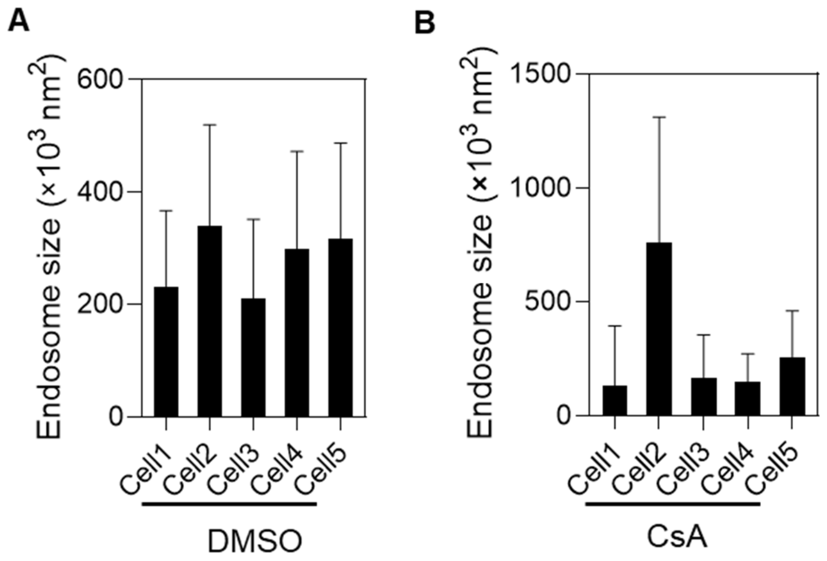


**Supplementary Figure S6.** Quantification of the average endosome size in control (DMSO treated) (**A**) and CsA treated (**B**) A549.IFITM3 cells, as in Figure 3. Data analyzed by 1 observer. Student's t-test for two-sample assuming equal variances was used, p-value based on two-tailed distribution.

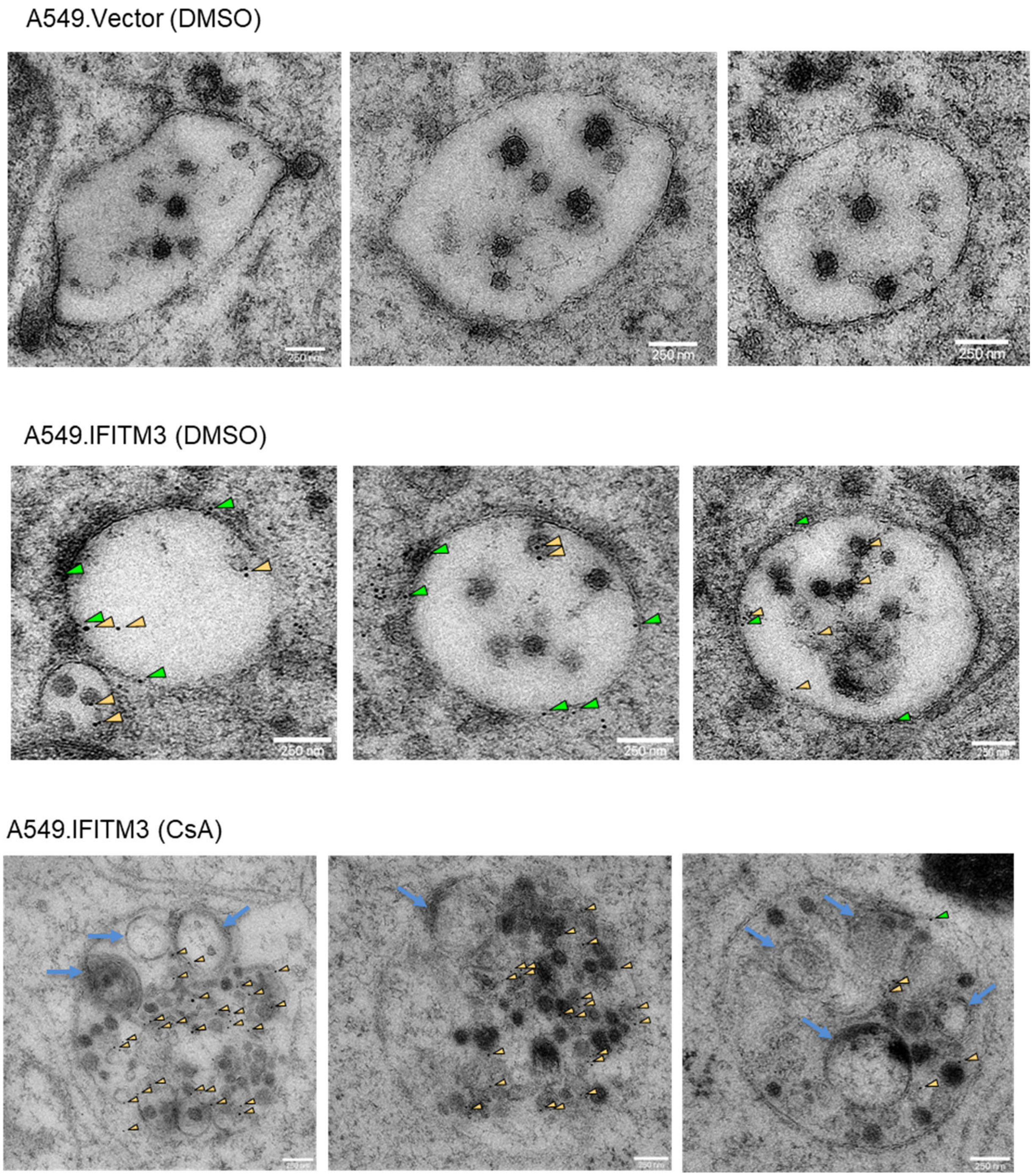


**Supplementary Figure S7.** Gallery of TEM images of resin sections from A549.Vector cells pretreated with DMSO (top panel), A549.IFITM3 cells treated with DMSO (middle panel), and A549.IFITM3 cells treated with CsA (lower panel). Gold nanoparticles located within the endosome's interior and limited membrane (LM) are indicated with melon and green arrowheads, respectively. Note the large, often multilamellar, inclusions in endosomes of CsA-treated cells (blue arrows). Scale bar is 250 nm.

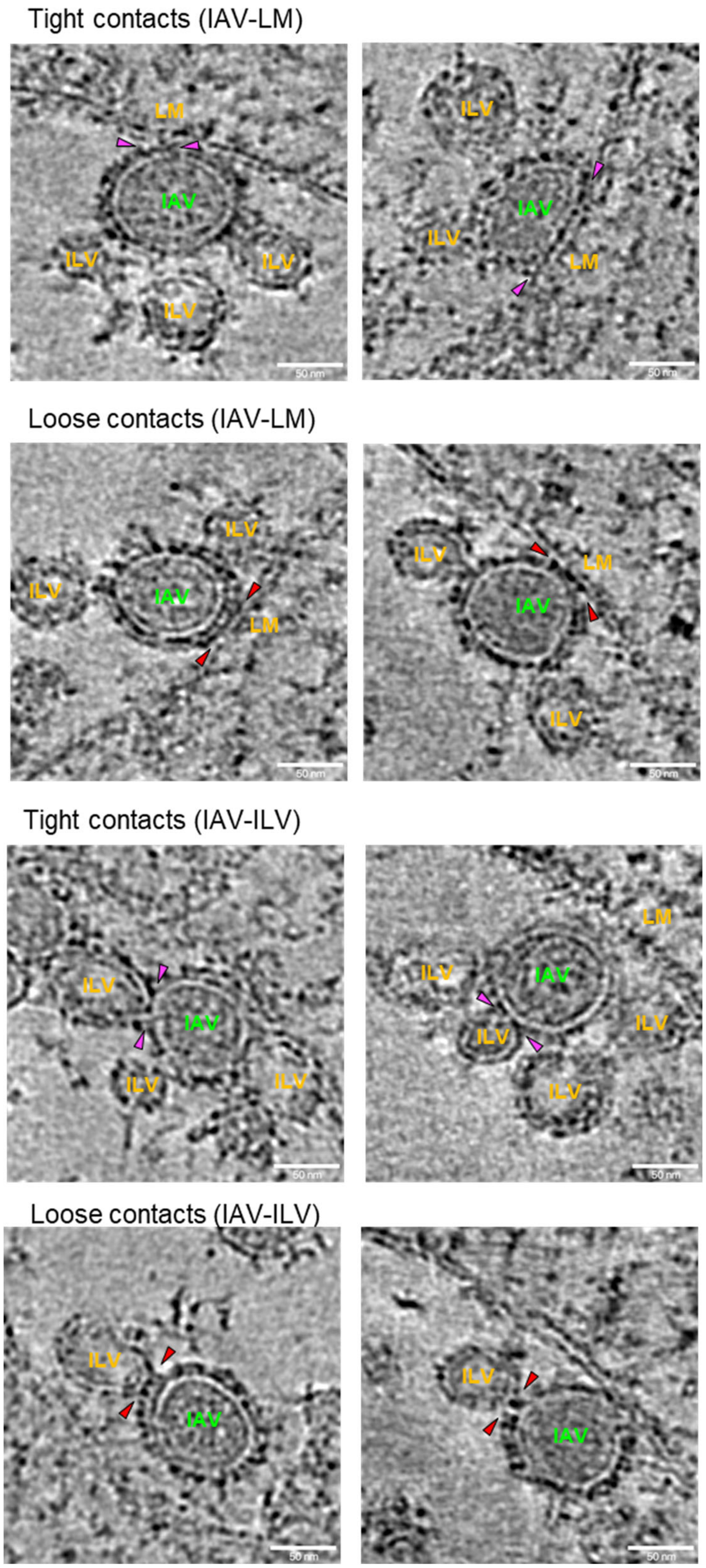


**Supplementary Figure S8.** A gallery of images showing examples of loose (red arrowheads) and tight (magenta arrowheads) contacts between IAV and LM, and between IAV and ILV, as indicated. Each image represents a 1.8 nm thick tomogram slice. Scale bar is 50 nm.

**Supplementary Video 1**

Reconstructed tomogram collected at representative endosome in DMSO-treated sample pictured in Figure 1. Each view represents a 10 nm thick slice. Scale bar is 250 nm.

**Supplementary Video 2**

A zoomed-in region of a reconstructed tomogram featuring a tight contact between IAV particle and endosomal LM shown in Figures 2A, S3A and S4A. Each view represents a 10 nm thick slice. Scale bar is 50 nm.

**Supplementary Video 3**

A zoomed-in region of a reconstructed tomogram featuring a hemifusion between IAV particle and ILV shown in Figures 2B, S3B and S4B. Each view represents a 10 nm thick slice. Scale bar is 50 nm.
